# 

**Published:** 1885-05-20

**Authors:** 


☞ **Notice.**--- Subscribers in arrears are requested to remit their dues at once. Please take due notice and govern yourselves accordingly.

### EDITORIAL NOTICES.

THE case of cerebro-spinal meningitis reported by Prof. Gaston in this number of the Journal terminated favorably. The concluding part of the article came in after we had gone to press, and will be published in our next.

R. L. POLK & CO., Publishers, Detroit, Mich., give notice that they are preparing to publish a *Medical and Surgical Dictionary* of the United States. It will contain a list of the Physicians in each State and Territory, information as to population, health, mortuary statistics, summary of state medical laws, list of medical journals, names of editors, medical colleges, hospitals, asylums, and much other information of interest to the medical Profession.

DEATH OF DR. JNO. H. LOGAN.—Dr. Logan, Professor of Chemistry in the Atlanta Medical College, died in this city on March 29th, 1885, of pulmonary disease, aged 65.

Professor Logan was a native of South Carolina. He did efficient service in the late war, both as a private soldier and in the medical department. Since the war, he resided in Alabama and in Georgia. He was elected to the Chair of Chemistry in Oglethorpe University and, subsequently, to the same chair in the Medical College of Atlanta. He was a man of ability as a teacher and writer, and an upright Christian gentleman.

### *RECEIPTED.*

1883.—W. A. Dobbs; T. L. Gibson.

1884.—J. B. Bailey. Joseph Jones to July, E. Y. Flemming, W. D. Hunt, W. H. Pool, J. A. Ardry to November, W B. Thomasson.

For 1885.—J. B. Burwick, W. H. Wilson to July, W. A. Shands, R. J. Talbert, J. H. Jennings, J. H. Boggans, Jno. G. Steed to May, J. F. Alexander, A. H. Smith to June, J. M. Bearden, A. D. Coleman, *A. Loveman, P. Simpson, Julius Butler, R. D. Selman, J. R. Rallston, P. P, Miller, G. J. Sloan, Thos. Law. Jas. G. Rush, W. T. Wilson, M. A. Chandler, R. C. Woodburry, T. A. Christian, James Baldy.*

